# Classification of Schizophrenia by Combination of Brain Effective and Functional Connectivity

**DOI:** 10.3389/fnins.2021.651439

**Published:** 2021-06-03

**Authors:** Zongya Zhao, Jun Li, Yanxiang Niu, Chang Wang, Junqiang Zhao, Qingli Yuan, Qiongqiong Ren, Yongtao Xu, Yi Yu

**Affiliations:** ^1^School of Medical Engineering, Xinxiang Medical University, Xinxiang, China; ^2^Engineering Technology Research Center of Neurosense and Control of Xinxiang city, Xinxiang, China; ^3^Engineering Technology Research Center of Neurosense and Control of Henan Province, Xinxiang, China; ^4^Xinxiang Key Laboratory of Biomedical Information Research, Henan Engineering Laboratory of Combinatorial Technique for Clinical and Biomedical Big Data, Xinxiang, China; ^5^School of International Education, Xinxiang Medical University, Xinxiang, China

**Keywords:** schizophrenia, effective connectivity, functional connectivity, classification, machine learning

## Abstract

At present, lots of studies have tried to apply machine learning to different electroencephalography (EEG) measures for diagnosing schizophrenia (SZ) patients. However, most EEG measures previously used are either a univariate measure or a single type of brain connectivity, which may not fully capture the abnormal brain changes of SZ patients. In this paper, event-related potentials were collected from 45 SZ patients and 30 healthy controls (HCs) during a learning task, and then a combination of partial directed coherence (PDC) effective and phase lag index (PLI) functional connectivity were used as features to train a support vector machine classifier with leave-one-out cross-validation for classification of SZ from HCs. Our results indicated that an excellent classification performance (accuracy = 95.16%, specificity = 94.44%, and sensitivity = 96.15%) was obtained when the combination of functional and effective connectivity features was used, and the corresponding optimal feature number was 15, which included 12 PDC and three PLI connectivity features. The selected effective connectivity features were mainly located between the frontal/temporal/central and visual/parietal lobes, and the selected functional connectivity features were mainly located between the frontal/temporal and visual cortexes of the right hemisphere. In addition, most of the selected effective connectivity abnormally enhanced in SZ patients compared with HCs, whereas all the selected functional connectivity features decreased in SZ patients. The above results showed that our proposed method has great potential to become a tool for the auxiliary diagnosis of SZ.

## Introduction

Schizophrenia (SZ) is one of the most severe and common mental disorders, and the World Health Organization (WHO) stated that more than 21 million people worldwide are affected by SZ. SZ is often accompanied by severe clinical symptoms including interrupted thinking and speech, hallucinations, and cognitive impairments (Van and Kapur, 2009; Haro et al., 2014), which usually lead to a significant reduction in the patients’ quality of life and bring huge economic burden to the society and family. Therefore, accurate diagnosis has important implications for subsequent treatment of SZ. Traditional clinical diagnosis of SZ mainly relies on interviews with patients by experienced psychiatrists, but this method is sometimes inaccurate and subjective (Andreas et al., 2009). Therefore, lots of researchers tried to use different techniques to identify objective and quantitative biomarkers of SZ, and these techniques were involved in immunology (Nurjono et al., 2012), genetics (Consortium, 2014), structural or morphological imaging (James et al., 2016; Pergola et al., 2016; Kubera et al., 2017; Woodward et al., 2018), functional imaging (Chyzhyk et al., 2015; Moghimi et al., 2018; Ji et al., 2019), and electroencephalography (EEG; Akar et al., 2016; Gomez-Pilar et al., 2017; Hunt et al., 2017; Devia et al., 2019). Compared with other methods, EEG still remains one of the most widespread techniques so far due to its high time resolution and low cost.

In recent years, researchers have reported the following hallmarks in SZ patients with the aid of EEG technique: abnormal event-related potential (ERP) amplitude/latency (Wang et al., 2003; Morris et al., 2008; Hamilton et al., 2017), altered spectral power of different frequency bands (Kam et al., 2013; BaAr et al., 2015; Hirano et al., 2015; Phillips and Uhlhaas, 2015; Hunt et al., 2017), and changes in complexity of EEG signal (Akar et al., 2015, 2016; Yu et al., 2016). However, most of the above EEG hallmarks were identified by using group-level statistical methods that do not provide a mechanism for classifying SZ at the individual level. Thus, some researchers have tried to use machine learning (ML) methods with these EEG biomarkers to differentiate SZ patients from healthy controls (HCs). Devia et al. (2019) used the ERP component evoked in a free-viewing paradigm as a feature and linear discriminant analysis (LDA) as a classifier to distinguish between SZ patients and HCs, and they reported an overall accuracy of 71%. Shim et al. (2016) used a combination of sensor-level (P300 amplitudes/latencies) and source-level (cortical current density values) EEG features during an auditory oddball task to train a support vector machine (SVM) classifier and finally got a high classification accuracy of 88.24%. Kim et al. (2015) tried to use delta band power to classify SZ patients but got a relatively poor classification accuracy of 62.2%. Bose et al. (2016) reported that when alpha band power during hyperventilation and post-hyperventilation was used as an input of a SVM classifier, a high classification accuracy of 83.33% was yielded. Chu et al. (2017) used EEG entropy during visual evocation of emotion as a feature and a SVM classifier to yield a relatively high classification accuracy of 81.5%. However, these studies seemed to have a common limitation: they usually used univariate indexes, i.e., spectral power/ERP/nonlinear measures of local electrodes or brain regions, as an input of a ML classifier.

It has been recently suggested that SZ could affect distributed brain neuronal network and induce a functional brain disconnection syndrome (Andreou et al., 2015a). A large number of EEG studies have confirmed dysfunctional connectivity in SZ patients at the group level (Andreou et al., 2015b; Di et al., 2015; Zhao et al., 2018). Thus, alterations in the brain connectivity patterns seem to serve as additional biomarkers of SZ. The so-called “brain functional connectivity” refers to the statistical dependence between two or more brain regions’ electrophysiological or other signals. Among the usually applied methods to study functional connectivity, such as spectral coherence and correlation coefficient, phase lag index (PLI), which allows for quantification of phase synchronization, has been widely applied in many studies because its most significant advantage is insensitivity to volume conduction effect as compared with other methods (Stam et al., 2007; Doesburg et al., 2013; Olejarczyk and Jernajczyk, 2017; Wang et al., 2017). The so-called “brain effective connectivity” that is another important dimension of functional connectivity can measure the directional flows of information among different brain areas. One of the most commonly used methods to construct effective connectivity is called partial directed coherence (PDC), which is a full multivariate spectral measure to determine the directed influences of Granger causality between any given signals in a multivariate set and has been successfully applied in measuring the multichannel directed cortical interactions (Silfverhuth et al., 2012; Sun et al., 2014a,b; Huang et al., 2016; Khandoker et al., 2019). Although altered brain connectivity patterns can provide useful information in discriminating SZ patients from HCs, it seems that only a few studies used EEG-based brain connectivity features as features to differentiate SZ. For example, Khandoker et al. (2019) used PDC effective connectivity as an input of a deep convolutional neural network (CNN) classifier to achieve a remarkable accuracy of 93.06%. However, brain functional connectivity and effective connectivity are two distinctly different methods and can reveal abnormal brain connectivity patterns of SZ from different aspects. Therefore, we hypothesize that the combination of brain functional connectivity and effective connectivity could provide a good discrimination between SZ patients and HCs.

In order to test the above hypothesis, ERP data were first recorded from SZ patients and HCs during a reinforcement learning task. Then, PLI and PDC methods were used to construct EEG-based brain functional and effective connectivity, respectively. Based on the constructed functional and effective connectivity, three strategies were tried to select appropriate brain connectivity features as an input of a supervised SVM classifier to classify SZ patients and HCs: SVM with only functional connectivity as features, SVM with only effective connectivity as features, and SVM with a combination of functional and effective connectivity. To the best of our knowledge, this is the first study using a combination of PDC effective connectivity and PLI functional connectivity as features for ML-based classification of SZ.

## Materials and Methods

### Electroencephalography Data

The EEG data used in our study were provided by Albrecht et al. (2016) and can be downloaded from an open access database called Zenodo (doi://10.5281/zenodo.29601 and doi://10.5281/zenodo.29064). The subjects, task, EEG recording, and preprocessing have been described in detail in Albrecht et al. (2016) and our recently published paper (Zhao et al., 2020). Therefore, we followed the methods of Zhao et al. (2020) in the following part. In brief, 45 SZ patients were recruited from the Maryland Psychiatric Research Center or other nearby clinics according to the Structured Clinical Interview for the *Diagnostic and Statistical Manual of Mental Disorders*, Fourth Edition (DSM-IV), and a group of 30 healthy subjects matched for sex and age was also recruited. For the experimental task, four image stimuli were presented to subjects (48 times/each image), and subjects were asked to respond by pressing a button (“Go”) or withholding a response (“NoGo”) to gain rewards (“Win,” monetary gain) or avoid punishments (“Avoid,” monetary loss). Therefore, four kinds of stimuli would appear throughout the experiment, i.e., “Go-to-Win,” “Go-to-Avoid,” “NoGo-to-Win,” and “NoGo-to-Avoid”; and subjects were asked to make the best response to win as much money as possible. For “Go-to-Win” and “NoGo-to-Win” stimuli, the subject was rewarded at a probability of 80% and got nothing (no reward and punishment) at a probability of 20% for a correct response; the subject got nothing at a probability of 80% and was rewarded at a probability of 20% for a wrong response. For “Go-to-Avoid” and “NoGo-to-Avoid” stimuli, the subject got nothing at a probability of 80% and punishment at a probability of 20% for a correct response; the subject got nothing at a probability of 20% and punishment at a probability of 80% for a wrong response. Monetary gain or loss was set at $0.05 each trial. The stimulus presentation sequence was as follows: a cross was presented for 400–600 ms, and then one of the four image stimuli was presented for 1,000 ms; next, a blank period was shown for 250–2,000 ms, and then a response period presented for 2,500 ms is indicated with an “O” for 1,500 ms; next a cross was presented for 1,000 ms; and finally, feedback image was shown for 2,000 ms. In this task, there were three types of feedback, i.e., positive feedback (thumb up image reflecting monetary gain), negative feedback (thumb down image reflecting monetary loss), and neutral feedback (thumb to the side image reflecting no monetary gain or loss). Here, we only used negative feedback-evoked ERPs. In their previously published paper, Albrecht et al. (2016) have described the procedure of EEG recording and preprocessing in detail, so we do not want to repeat them here. In a word, 32 channel EEG data were recorded during the above task, re-referenced to linked mastoids and down-sampled to 256 Hz. After standard EEG preprocessing process, the cleaned negative feedback-locked ERPs [(-1,500, 1,500) ms around negative feedback stimulus, baseline corrected to (-1,000) ms] were obtained for further analysis. In addition, 20 EEG channels (FP1, FP2, Fz, F3, F4, F7, T7, T8, C3, C4, Cz, Pz, P4, P3, F8, P8, P7, Oz, O2, and O1) were selected in our study.

### Time-Frequency Power Calculation

Time-frequency power calculation of a single trial data was performed with Morlet wavelet transform where peak frequency ranging from 3.9 to 40 Hz and cycle number varying from 3 cycles at 3.9 Hz to 11.4 cycles at 40 Hz were linearly divided into 50 points, respectively. Finally, for every trial data, we obtained a 2D matrix of 50 (frequency points) × 768 (time points).

### Phase Lag Index Functional Connectivity Feature

Phase lag index, a phase synchronization index, was used for quantification of functional connectivity between each EEG channel pair in the present study (Stam et al., 2007):

(1) $$P\,L\,I_{\left(f,t\right)}=\left|\frac{1}{M}\,\sum_{m=1}^{M}s\,g\,n\,\left(△\,\varphi_{a,b}^{m}\,\left(f,t\right)\right)\right|$$

where $△\,\varphi_{a,b}^{m}\,\left(f,t\right)$ stands for phase difference between channel *a* and *b* at frequency *f* and time *t* of trial *m*, *M* stands for the number of trials, and *sgn* indicates the sign (−1 for negative values, +1 for positive values, and 0 for zero values).

With the use of the above equation, a 20 × 20 PLI functional connectivity matrix could be obtained at each time-frequency point. In the present study, PLI values were averaged over theta band (4–7 Hz) and a time period from 0.1 to 0.6 s after stimulus onset to obtain a weighted 20 × 20 functional connectivity matrix used as candidate feature for each subject.

### Partial Directed Coherence Effective Connectivity Feature

In this study, PDC was used to determine the directed influences among these 20 channel EEG signals. Detailed descriptions and calculation methods can be found in some previously published literatures (Sun et al., 2014a,b). In brief, let *X*(*n*) = [*x*_1_(*n*), *x*_2_(*n*), *x*_3_(*n*), *…*, *x*_*N*_(*n*)]*^*T*^* stand for EEG signal of *N* channels (*N* = 20 here), and then a multivariate autoregressive (MVAR) model can be used to describe *X*(*n*):

(2) $$X\,\left(n\right)=\sum_{r=1}^{p}A_{r}\,X\,\left(n-r\right)+W\,\left(n\right)$$

where *W*(*n*) is a multivariate uncorrelated noise vector, *A*_*r*_ is the coefficient matrix, and *p* is the MVAR model order, which can be measured by using the Bayesian information criterion (BIC). In the current study, an optimal order of 9 was used. The *A*_*r*_ can be computed by using Yule–Walker equations:

(3) $$\sum_{r=1}^{p}A_{r}\,R\,\left(-k+r\right)=0$$

where *R*(*m*) = < *X*(*n*) *X*^*T*^(*n* + *m*)> are the covariance matrices of all *X*(*n*) with lag *m*, *k* = 1, 2, …, *p.* The Levinson–Wiggins–Robinson (LWR) algorithm is one of the most used methods to determine *A*_*r*_(Morf et al., 1978). For ERP data, many trials of the same process are available, and a modified adaptive procedure for estimating the MVAR model can be applied (Ding et al., 2000). Briefly, the covariance matrices *R*_*n*_(*m*) (*n* = 1, 2, …, *T*) for *T* trials were computed to obtain the averaged covariance matrix ($\bar{R}\,\left(m\right)=\,\sum_{n=\,1}^{T}R_{n}\,\left(m\right)/T$) for each subject, then the *R*(*m*) in Eq (3) was replaced with $\bar{R}\,\left(m\right)$, and finally, the *A*_*r*_ was determined.

After the MVAR model is estimated, the PDC value from channel *j* to channel *i* at frequency *f* can be computed as follows:

(4) $$\text{PDC}\,\left(i,j,f\right)=\frac{\left|A_{i\,j}\,\left(f\right)\right|}{\sqrt{\sum_{k}A_{k\,j}^{*}\,\left(f\right)\,A_{k\,j}\,\left(f\right)}}$$

where *A*(*f*) stands for the difference between the *N*-dimensional identity matrix and the Fourier transform of *A*_*r*_, * stands for matrix transposition and complex conjugation, and *A*_*ij*_(*f*) are the elements of the matrix *A*(*f*).

A nonparametric method based on surrogate data was applied here to test the significance of PDC value (Astolfi et al., 2005). In brief, the phase of the Fourier coefficients of original EEG signals from each epoch was randomly shuffled, and the inverse Fourier transform was performed to obtain a surrogate data. Then, the obtained surrogate data were used to re-compute PDC. After the above-mentioned shuffling procedure were repeated 5,000 times, an empirical distribution of PDC values could be obtained. Based on this empirical distribution, a significance threshold of *p* = 0.05 was used to determine if the PDC value was a real connection.

In this study, we used time window of (0.1, 0.6) s after stimulus onset to compute PDC, and the obtained PDC(*i*,*j*,*f*)value was averaged over the theta band (4–7 Hz) to obtain a 20 × 20 PDC effective connectivity matrix used as a candidate feature for each subject.

### Feature Selection and Classification of Schizophrenia

Until now, we obtained a total of 570 features including 20 × (20 − 1)/2 = 190 functional connectivity features and 20 × (20 − 1) = 380 effective connectivity features. It is undesirable to use all the features that usually contain lots of irrelevant features for direct classification, because these irrelevant features will lead to a bad classification performance. In order to select relevant and key features for SZ classification, Fisher score was used as a feature selection criterion, which has been widely used in many previously published classification studies (Hwang et al., 2014; Zhang et al., 2015; Shim et al., 2016). A higher Fisher score means that this feature has a higher discriminative power than a feature with a lower Fisher score. Based on the Fisher score, three strategies were implemented to select appropriate brain connectivity features: (1) only functional connectivity features: for functional connectivity feature set, the top *N* features with the highest Fisher scores were selected for classification, and *N* varies from 1 to 50 to find optimal feature number. (2) Only effective connectivity features: for effective connectivity feature set, the top *M* features with the highest Fisher scores were selected for classification, and *M* also varies from 1 to 50 to search optimal feature number. (3) A combination of functional and effective connectivity features: the total number of features was set to *L* varying from 2 to 50, which contained the top *n* and *m* (*L* = *n* + *m*) features with the highest Fisher scores from functional and effective connectivity feature set, respectively. In order to search the optimal combination of these two kinds of features, *n* varies from 1 to *L* − 1, while *m* changed from *L* − 1 to 1.

The classification of SZ patients and HCs was performed using an SVM classifier with a linear kernel function. The SVM classifier used in the present study was provided by LIBSVM toolbox (Chang and Lin, 2011). The classification accuracy (ACC), specificity (SPE), sensitivity (SEN), and area under the receiver operating characteristic (AUC) curve were determined using the leave-one-out cross-validation (LOOCV).

### Statistics

In the present study, we performed the comparison of time-frequency power maps between SZ patients and HCs, which involved a total of 15,350 comparisons (307 time points from −0.2 to 1.0 s, 50 frequency points from 3.9 to 40 Hz). Thus, we applied a cluster-based nonparametric permutation test to control multiple comparison problem (Maris and Oostenveld, 2007). Briefly, 10,000 random permutations of the time-frequency power maps between two groups were performed. For each permutation, *t*-test was carried out between the permuted time-frequency maps, all pixels with an uncorrected *p* value below 0.01 were formed into clusters, and the largest cluster (i.e., the cluster with the largest number of pixels) was stored. Therefore, we obtained a null distribution of the largest clusters after 10,000 random permutations. Finally, the original time-frequency maps of two groups were also compared using *t*-test, all pixels with *p* values below 0.01 were formed into clusters, and any clusters that were less than the (1 − α) percentile of the null distribution of largest clusters expected were removed. In our current study, the family-wise α level was set to 0.05.

The nonparametric permutation test is one of the most popular methods to access the statistical significance of the classification performance (Golland and Fischl, 2003; Ojala and Garriga, 2010). However, traditional permutation test for accessing how likely the observed classification ACC would be obtained by chance usually determines the null distribution by permuting the labels in the data. Therefore, a modified permutation test procedure that was proposed by Ojala and Garriga (2010) was applied in the present study. In brief, a randomized version of training data was obtained by applying independent permutation to the elements of each feature of the original training data, and then the LOOCV was performed on the permutated training data to produce a classification accuracy (denoted as ACC_*rand*_). After the above process was repeated *N* times (*N* = 10,000 in current study), an empirical distribution of the ACC_*rand*_ under null hypothesis was obtained. The *p* value of nonparametric permutation test was defined as the ratio of the number of ACC_*rand*_ in the null distribution that was larger than the ACC obtained on the original training data to the total number of permutations.

In addition, the comparison of connectivity strength between two groups was carried out using nonparametric Wilcoxon signed rank test with false discovery rate (FDR) correction for controlling multiple comparison problem (Genovese et al., 2002).

## Results

### Time-Frequency Analysis

**FIGURE 1:**
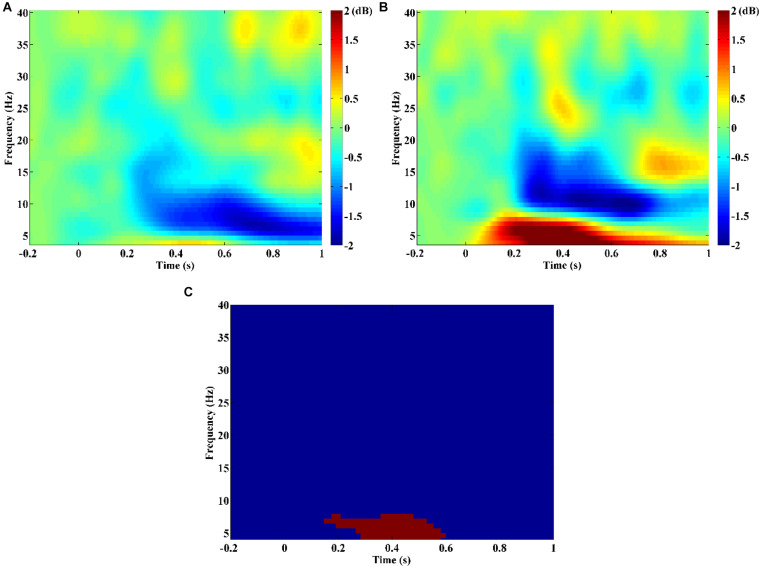
Time-frequency power maps for **(A)** schizophrenia (SZ) and **(B)** healthy controls (HCs). Time-frequency power maps were averaged over two channels (F3, F4) and plotted as 10log10 change over baseline (from −0.2 to −0.1 s). **(C)** The result of the statistical comparison. Red color indicates time–frequency regions with significant difference between two groups, and blue color indicates no significant difference.

The time-frequency analysis result showed that SZ patients exhibited obvious theta/alpha band desynchronization, which began to occur at about 0.2 s after stimulus onset (Figure 1A). Unlike SZ patients, HCs showed obvious theta band synchronization that occurred immediately after stimulus onset and alpha/beta band desynchronization that started to occur at about 0.2 s after stimulus onset (Figure 1B). Statistical comparison between the two groups indicated that theta band power of HCs was stronger compared with that of SZ during the time window from about 0.17–0.6 s after stimulus onset (Figure 1C, *p* = 0.003). Therefore, it was reasonable to compute PDC and PLI brain connectivity as classification features over the theta band and time period of 0.1, 0.6 s after stimulus onset in our current study.

### Brain Functional and Effective Connectivity Patterns

**FIGURE 2:**
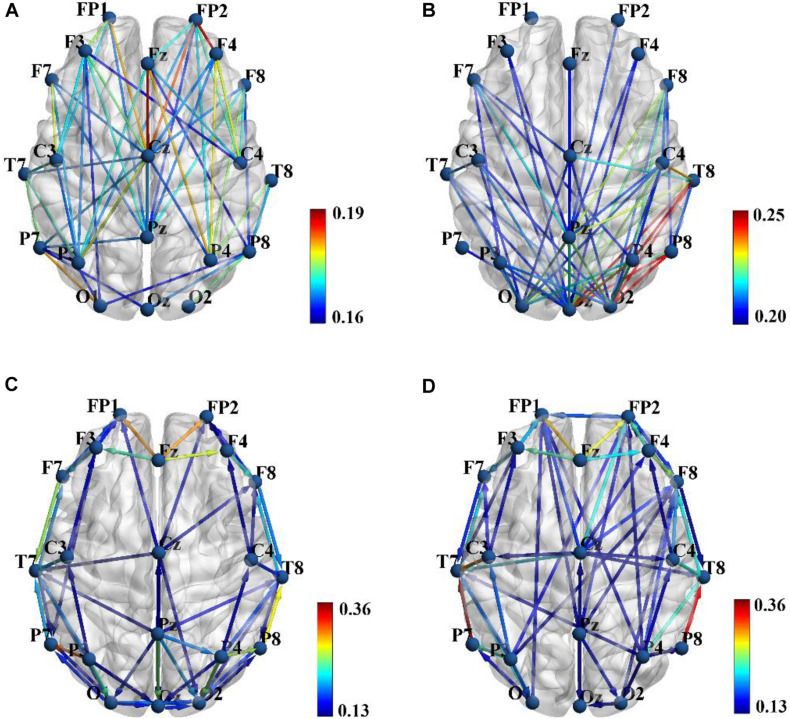
Group average phase lag index (PLI) functional connectivity pattern for schizophrenia (SZ) patients **(A)** and healthy controls (HCs) **(B)**, and sparsity was set to 0.3 to obtain a better illustration. Group average partial directed coherence (PDC) effective connectivity pattern for SZ patients **(C)** and HCs **(D)**, and sparsity was set to 0.15 to obtain a better illustration. Color bar shows the connectivity strength. Brain connectivity was visualized with the BrainNet Viewer toolbox (http://www.nitrc.org/project/bnv/).

Figures 2A,B show the group average PLI functional connectivity pattern of SZ patients and HCs, respectively, and we qualitatively observed that the main difference between two groups was that SZ patients exhibited much less and much weaker long-range connections between the frontal/temporal cortexes and the visual cortex than did HCs. However, the PDC effective connectivity pattern seemed to reveal the abnormal connections of SZ patients from another aspect, and we found that HCs showed obvious long-range down-top information flows from the parietal to frontal regions (Figure 2D), whereas there was almost no such directional information transmission for SZ patients (Figure 2C).

### Classification Result

**FIGURE 3:**
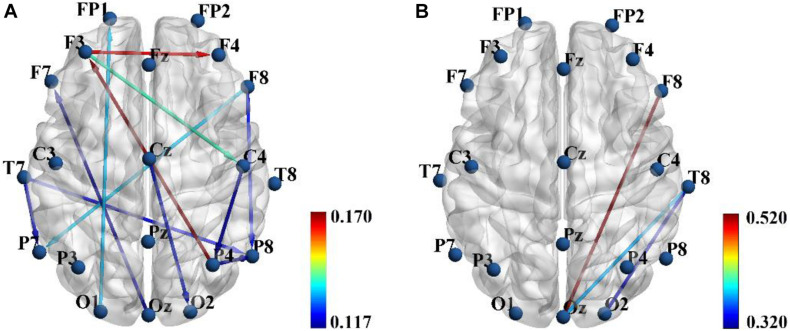
The spatial distribution of the 12 partial directed coherence (PDC) **(A)** and three phase lag index (PLI) **(B)** connectivity features selected from the combined feature set when the classification performance was optimal. Color bar represents the discriminative power i.e., Fisher score.

In the present study, three feature sets were tested in order to get a good discrimination between SZ patients and HCs: (1) only functional connectivity features, (2) only effective connectivity features, and (3) a combination of functional and effective connectivity features. We repeated the LOOCV with a varying number of features. Table 1 shows the obtained best performance with the corresponding feature numbers. As shown in Table 1, a good classification performance (SPE = 88.89%, SEN = 76.92%, AUC = 0.825, and ACC = 83.87%, *p* < 0.0001) was achieved with feature number of 6 when using only PLI functional connectivity feature set. A better classification performance (SPE = 86.11%, SEN = 88.46%, AUC = 0.909, and ACC = 87.97%, *p* < 0.0001) was obtained with feature number of 14 when using only PDC functional connectivity. However, we obtained the best classification performance (SPE = 94.44%, SEN = 96.15%, AUC = 0.952, and ACC = 95.16%, *p* < 0.0001) when using the combination of PLI and PDC connectivity features, and the corresponding selected feature number was 15, which included 12 PDC and three PLI connectivity features. The above results indicated that the combination of functional and effective connectivity features could provide a better discrimination between SZ patients and HCs than single functional or effective connectivity. The spatial distribution of the selected 15 connectivity features (12 PDC and three PLI connectivity features) is shown in Figure 3. As shown in Figure 3A, the 12 PDC connectivity features with the highest discriminative power mainly contained four kinds of effective connectivity pattern: (1) long-range “top-down” and “down-top” interactions between frontal cortex and occipital/parietal cortexes (F8→P7, F8→P8, P4→F3, O1→FP1, and Oz→F7); (2) top-down links from the temporal/central cortexes to the visual/parietal cortexes (T7→P7, T7→P8, Cz→O2, and C4→P4); (3) top-down information flow from the frontal cortex to the central cortex (F3→C4); and (4) local information flows in the frontal and parietal cortexes (F3→F4 and P4→P8). Interestingly, the three selected PLI connectivity features with the highest discriminative power showed an asymmetric right hemisphere lateralized pattern (Figure 3B) and mainly exhibited long-range connections between the frontal/temporal cortexes and the visual cortex (F8-Oz, T8-Oz, and T8-O2).

**TABLE 1:** The obtained best classification performance (ACC, SPE, SEN, and AUC) with the corresponding feature numbers on three different feature sets.

Feature set	Best classification performance				Feature number
	
	ACC (%)	SPE (%)	SEN (%)	AUC	
PLI connectivity	83.87	88.89	76.92	0.825	6
PDC connectivity	87.97	86.11	88.46	0.909	14
Combination of PLI and PDC connectivity	95.16	94.44	96.15	0.952	15 (12 effective and 3 functional connectivity)

*ACC, classification accuracy; SPE, specificity; SEN, sensitivity; AUC, area under the receiver operating curve; PLI, phase lag index; PDC, partial directed coherence.*

Table 2 summarizes the values of the selected 12 PDC and three PLI connectivity features and the results of statistical comparison between SZ patients and HCs. As shown in Table 2, SZ patients exhibited reduced down-top information flows from the occipital and parietal cortexes to the frontal cortex (P4→F3, O1→FP1, and Oz→F7) than did HCs. As for other PDC effective connectivity features, SZ patients showed abnormally enhanced values as compared with HCs. More interestingly, we found that SZ patients exhibited significantly decreased phase synchronization between the frontal/temporal cortexes and the visual cortex (F8-Oz, T8-Oz, and T8-O2) than did HCs. The result of statistical comparison implied that all the 15 selected brain connectivity features had statistical difference between two groups, which proved that the proposed feature selection method in our study could reliably find the connectivity with high discriminative power.

**TABLE 2:** The brain connectivity values of the selected 12 PDC and three PLI connectivity features and the result of statistical comparison between SZ patients and HCs.

Brain connectivity	Strength (mean ± SEM)			*p* (FDR corrected)
	
	SZ	HC		
P4→F3	0.015 ± 0.011	0.094 ± 0.020	**↓**	<0.001
F3→F4	0.057 ± 0.015	0.004 ± 0.004	**↑**	0.011
F3→C4	0.047 ± 0.014	0.000 ± 0.000	**↑**	0.011
F8→P7	0.052 ± 0.016	0.002 ± 0.002	**↑**	0.015
O1→FP1	0.043 ± 0.015	0.139 ± 0.033	**↓**	0.011
F8→P8	0.040 ± 0.013	0.000 ± 0.000	**↑**	0.011
Cz→O2	0.150 ± 0.032	0.044 ± 0.021	**↑**	0.019
T7→P8	0.036 ± 0.011	0.003 ± 0.003	**↑**	0.013
T7→P7	0.123 ± 0.031	0.028 ± 0.014	**↑**	0.014
P4→P8	0.253 ± 0.034	0.128 ± 0.032	**↑**	0.014
C4→P4	0.107 ± 0.025	0.029 ± 0.013	**↑**	0.032
Oz→F7	0.041 ± 0.015	0.110 ± 0.022	**↓**	0.011
F8-Oz	0.145 ± 0.007	0.223 ± 0.013	**↓**	<0.001
T8-Oz	0.149 ± 0.009	0.247 ± 0.020	**↓**	<0.001
T8-O2	0.147 ± 0.009	0.245 ± 0.022	**↓**	<0.001

*“↓” and “↑” indicate that the connectivity strength of SZ patients is lower and higher than that of HCs, respectively.*

*SEM, standard error of mean; PDC, partial directed coherence; PLI, phase lag index; SZ, schizophrenia; HCs, healthy controls; FDR, false discovery rate.*

## Discussion

In this study, we proved that simultaneous application of brain functional and effective connectivity could greatly improve overall classification performance in the ML-based discrimination of SZ. An excellent classification ACC of 95.16% was obtained when the combination of functional and effective connectivity feature was used, which was much higher than either the functional connectivity feature set (83.87%) or the effective connectivity feature set (87.97%). Studying the 15 connectivity features (12 PDC and three PLI connectivity features) selected from the combined feature set implied that brain effective and functional connectivity could reveal altered brain connectivity pattern of SZ from different aspects: all the down-top information flows from the occipital/parietal cortexes to the frontal cortex (P4→F3, O1→FP1, and Oz→F7) were reduced, whereas other effective connectivity was enhanced in SZ patients compared with HCs, and all the three PLI functional connectivity was reduced in SZ patients.

### Advantages of Combination of Functional and Effective Connectivity for Classification

Different from brain functional connectivity that refers to the statistical dependence between two brain regions’ electrophysiological signals and usually does not imply the existence of causal influence of one region on another, brain effective connectivity measures the directional information flow among different brain areas and can be computed by a method based on Granger causality. Therefore, functional or effective connectivity can only capture one different aspect of interdependence, and their combination could provide complementary information. Actually, some previous studies have proved this (Dauwels et al., 2010; Blinowska et al., 2017). For example, Dauwels et al. (2010) computed more than 30 EEG-based measures of functional and effective connectivity, and they found that many of those measures were strongly correlated and seemed to provide little complementary information about EEG synchrony. They further observed that only Granger causality measure and some functional connectivity measures including phase synchrony indices were mutually uncorrelated, and the combination of Granger causality measure and functional connectivity was used as features to distinguish mild cognitive impairment (MCI) patients from HCs, yielding a relatively high classification ACC of 83%. Blinowska et al. (2017) demonstrated that the use of both effective and functional connectivity could significantly increase classification performance of Alzheimer’s disease (AD) individuals from HCs as compared with when only effective or functional connectivity was used.

The PLI functional connectivity pattern analysis showed that SZ patients exhibited much less and much weaker long-range connections between the frontal/temporal cortexes and the visual cortex than did HCs (Figures 2A,B), while PDC effective connectivity pattern analysis implied that there were almost no long-range down-top information flows from the parietal to frontal regions for SZ patients compared with HCs (Figures 2C,D). Therefore, we believed that the combination of effective and functional connectivity could provide complementary information about the altered brain connectivity pattern of SZ patients. However, to the best of our knowledge, the combination of functional and effective connectivity has not been used as features for ML-based classification of SZ. Our further result based on SVM classification indicated that the simultaneous application of brain functional and effective connectivity could greatly improve overall classification performance in the ML-based discrimination of SZ, which was consistent with the results of previous studies (Dauwels et al., 2010; Blinowska et al., 2017).

### Altered Theta-Band Functional and Effective Connectivity in Schizophrenia Patients

Our results of time-frequency analysis revealed that only theta-band oscillatory activity of HCs was significantly stronger than that of SZ patients after negative feedback stimuli (Figure 1). Actually, a large number of studies have found that increased theta activity was often observed in SZ patients during the rest state whereas reduced theta activity in SZ patients during various cognition tasks in recent years (Moran and Hong, 2011; Başar and Güntekin, 2013). Therefore, we have enough reasons to use theta-band effective and functional connectivity as features to classify SZ patients from HCs in this study.

Our classification result based on the SVM classifier finally selected 15 brain connectivity features (12 PDC and three PLI connectivity features) with the highest discriminative power (Figure 3 and Table 2). The 12 PDC effective connectivity features were mainly located between the frontal/temporal/central lobes and visual/parietal lobes (Figure 3A), and the three PLI functional connectivity features were mainly located between the frontal/temporal cortexes and visual cortex of the right hemisphere (Figure 3B). Li et al. (2019) used phase locking value (PLV) to study the EEG functional connectivity of SZ patients during a P300 task and observed that long-range functional connections including the frontal-occipital, temporal-occipital, and frontal-parietal connectivity were significantly reduced in SZ patients compared with HCs. Ganella et al. (2017) used functional magnetic resonance imaging (fMRI) technique and found that functional connectivity strength predominantly involving fronto-temporal, fronto-occipital, and temporo-occipital connections were reduced in SZ patients. These previous results seemed to be consistent with our findings that the selected three PLI functional connectivity including frontal-occipital and temporal-occipital connections were significantly decreased in SZ patients. However, for the effective connectivity, we did not found any similar researches that studied the abnormal effective connectivity pattern in SZ patients during a cognitive task. Therefore, similar studies from other groups were needed to evaluate the consistency of our results of the PDC effective connectivity in the future.

### Compared With Previous Studies

The comparison of the classification result in our study with that of previous EEG studies is illustrated in Table 3. As shown in Table 3, it was obvious that the classification accuracy obtained in this work was better than that of other studies except the literature (Oh et al., 2019) that obtained an excellent classification accuracy of 98.07% by applying an 11-layered deep CNN model to extract features automatically. A direct comparison of the present classification results with those of previous related studies was difficulty because different classifiers were used in different studies. However, when compared with the studies that also used the SVM classifier (Bose et al., 2016; Santos-Mayo et al., 2016; Shim et al., 2016; Chu et al., 2017; Jahmunah et al., 2019; Li et al., 2019), it was evident that our study exhibited the highest accuracy. We believed that the excellent classification performance in our study was mainly due to the simultaneous use of effective and functional connectivity as features that could reveal the abnormal brain alterations of SZ more comprehensively.

**TABLE 3:** Comparison of the classification accuracy of SZ patients with other previous studies.

Number of subjects	Feature set	Classifier	Best classification accuracy (%)	References
				
34 SZ patients and 34 HCs	Combined sensor-level P300 amplitude and source-level current density	SVM	88.24	Shim et al., 2016
14 SZ patients and 14 HCs	Nonlinear measures	SVM	92.91	Jahmunah et al., 2019
45 SZ patients and 39 HCs	PDC effective connectivity and graph topological measures	Multi-domain connectome CNN	93.06	Phang et al., 2019
16 SZ patients and 31 HCs	P300 amplitude and latency	SVM	92.23	Santos-Mayo et al., 2016
34 SZ patients and 10 HCs	EEG entropy during visual evocation of emotion	SVM	81.50	Chu et al., 2017
14 SZ patients and 14 HCs	Features are extracted automatically	11-layered deep CNN	98.07	Oh et al., 2019
11 SZ patients and 9 HCs	ERP amplitude during scene free-viewing	LDA	71.00	Devia et al., 2019
23 SZ patients and 25 HCs	Combined SPN features of the rest and task networks	SVM	90.48	Li et al., 2019
57 SZ patients and 24 HCs	Alpha band power	SVM	83.33	Bose et al., 2016
90 SZ patients and 90 HCs	Delta band power	ROC analysis	62.20	Kim et al., 2015
24 SZ patients and 24 HCs	Amplitudes/latencies of N100 and P300 during an auditory oddball task	KNN	72.40	Neuhaus et al., 2013
45 SZ patients and 30 HCs	Combination of PDC effective and PLI functional connectivity	SVM	95.16	Present study

*SPN, spatial pattern of network; SVM, support vector machine; CNN, convolutional neural network; LDA, linear discriminant analysis; ROC, receiver operating characteristic; KNN, K-nearest neighbor; SZ, schizophrenia; HCs, healthy controls; PDC, partial directed coherence; EEG, electroencephalography; ERP, event-related potential; PLI, phase lag index.*

### Limitations and Future Directions

Although the classification performance in the present study using a combination of effective and functional connectivity is encouraging, there are still some limitations that should be considered in the future. Firstly, the sample size of subjects in the present study is relatively small, and therefore a large independent dataset should be used to test our methods and confirm the results. Secondly, all the SZ patients were on medication, and thus, we could not control for possible confounding effects of the drugs. Moreover, our method in the present study is effective by using “task-state” effective and functional connectivity as features and only applied for the discrimination of SZ; therefore, it is necessary to test our method on “resting-state” EEG data and on other psychiatric disorders to prove its generalizability in the future.

## Conclusion

In this study, a combination of brain effective and functional connectivity was proposed for ML-based discrimination of SZ. Results indicated that brain effective and functional connectivity analysis could reveal the altered brain connectivity pattern of SZ from different aspects, and the simultaneous use of PDC effective and PLI functional connectivity features could reliably differentiate SZ patients from HCs with a high classification accuracy of 95.16%, a specificity of 94.44%, and a sensitivity of 96.15%, which was better than most of the previously reported results.

## Data Availability Statement

The datasets presented in this study can be found in online repositories. The names of the repository/repositories and accession number(s) can be found below: https://zenodo.org/record/29601.

## Ethics Statement

The studies involving human participants were reviewed and approved by University of Maryland Institutional Review Board. The patients/participants provided their written informed consent to participate in this study.

## Author Contributions

ZZ carried out the effective and functional connectivity computation and ML-based classification and wrote the first draft of the manuscript. JL and CW carried out the time-frequency power analysis. YY, QR, and QY undertook the statistical analysis. YX and JZ modified the manuscript. YN helped answer the reviewer’s questions, modify the manuscript according to the reviewers’ comments and check the proof. All authors contributed to the article and approved the submitted version.

## Conflict of Interest

The authors declare that the research was conducted in the absence of any commercial or financial relationships that could be construed as a potential conflict of interest.
